# Design and Fabrication of a Liver-on-a-chip Reconstructing Tissue-tissue Interfaces

**DOI:** 10.3389/fonc.2022.959299

**Published:** 2022-08-05

**Authors:** Jing Liu, Chong Feng, Min Zhang, Feng Song, Haochen Liu

**Affiliations:** ^1^ School of Biology, Food and Environment, Hefei University, Hefei, China; ^2^ Shandong Key Laboratory of Biophysics, Institute of Biophysics, Dezhou University, Dezhou, China; ^3^ Department of Cardiovascular Surgery, Xi’an Children’s Hospital, Xi’an, China

**Keywords:** tissue-tissue interfaces, vascularized liver tissue, substance concentration gradient, bilayer microspheres, organ-on-chip

## Abstract

Despite the rapid advances in the liver-on-a-chip platforms, it remains a daunting challenge to construct a biomimetic liver-on-a-chip for *in vitro* research. This study aimed to reconstruct the tissue-tissue interfaces based on bilayer microspheres and form vascularized liver tissue. Firstly, we designed a tri-vascular liver-on-a-chip (TVLOC) comprising a hepatic artery, a portal vein and a central vein, and theoretically analyzed the distribution of velocity and concentration fields in the culture area. Secondly, we designed a bilayer microsphere generating microsystem based on the coaxial confocal principle, which is primarily used to produce bilayer microspheres containing different kinds of cells. Finally, the bilayer microspheres were co-cultured with endothelial cells in the cell culture area of the TVLOC to form vascularized liver tissue, and the cell viability and vascular network growth were analyzed. The results revealed that the TVLOC designed in this study can provide a substance concentration gradient similar to that of the liver microenvironment, and the bilayer microspheres can form a three-dimensional (3D) orderly liver structure with endothelial cells. Such a liver-on-a-chip is capable of maintaining the function of hepatocytes (HCs) pretty well. This work provides full insights into further simulation of the liver-on-a-chip.

## Introduction

Drug development is costly and typically suffers from colossal failure during clinical trials. This is partially due to the fact that animal models fail to precisely predict the efficacy and safety of drugs in humans (1, 2). Thus, recapitulating the complex tissue structures *in vitro* according to their native morphologies is significant to drug screening and toxicity testing. Advances in microengineering-related technologies have opened up brand-new possibilities for creating *in vitro* models, being capable of reconstructing more complex 3D *in vitro* models and incorporating vital dynamic mechanical and chemical factors (3). Especially, micro-fluidic perfusion systems have emerged as a promising platform for the culture of such engineered tissues offering the ability to properly supply nutrients, oxygen, and growth factors, accurately control the flow of fluid, as well as timely remove waste (4), and have been demonstrated to facilitate the remarkable advance of organs-on-chips (5–7). Among various organs, the liver, as the largest inner organ of the human body, exerts a central function in drug metabolism and detoxification, which is critical to survival and cannot be compromised. Drug-induced hepatotoxicity or drug-induced liver injury (DILI) is the primary reason for discontinuing clinical trials and for drug withdrawal from the market, thus highlighting a pressing need for developing *in vitro* liver tissue models for drug screening and evaluation (8). Most recently, with the accumulating popularity of organs-on-chips, liver-on-a-chip, particularly, is highly expected to become a promising alternative to animal models (9, 10).

Liver lobules, as the basic structural unit of the liver, are typically hexagonal and each of them is supplied with dual blood vessels. The hepatic artery provides blood rich in oxygen, and the hepatic portal vein provides blood rich in nutrients but low in oxygen. These two types of blood converge in the liver sinusoids between the hepatocytes (HCs) and then flow out from the central vein. The hyperoxia blood in the hepatic artery merges with the hypoxic blood in the hepatic portal vein and brings about oxygen concentration gradients, which promotes the formation of liver zonation (11). Liver zonation is characterized by regional changes in hepatocyte function along the direction of liver sinusoid. The heterogeneity of the liver region enables the liver to maintain stable blood glucose levels during carbohydrate metabolism, whether during eating or fasting (12). Due to differences in gene expression and microenvironment regions, the adverse effects of drugs or toxins (such as carbon tetrachloride) are region-specific in the liver. The concentration gradients of oxygen, hormones, glucose and extracellular matrix are regarded as the main regulators for the formation of heterogeneous regions (13). In a static culture system, liver cells are exposed to a homogeneous environment of oxygen and hormone, which cannot simulate the concentration gradient of substances *in vivo*. Accordingly, the realization of physiological substance concentration gradients in the microsystem is conducive to further elucidating the regulatory functions of the liver and the role of oxygen in normal pathophysiological processes. Currently, the liver-on-a-chip has made tremendous progress, and the structure and function thereof are getting closer to that of the liver *in vivo*. Many a researcher has been dedicated themselves to fabricating a robust liver-on-a-chip platform. Banaeiyan et al. (14) developed a lobule-like bioinspired liver-on-a-chip *in vitro* microdevice composed of two independent layers, with the bottom layer carrying the culture chambers and diffusion channels and the top layer possessing a dual seed-feed functionality. This tissue-like hexagonal architecture and diffusion channels recreated the convection-diffusion of blood circulation in the liver. Plus, 3D tissue-like structure and bile-canaliculi network formation were observed in the chips. In a study by Du et al. (15), an *in vitro* liver sinusoid chip was developed by integrating murine primary HCs, liver sinusoidal endothelial cells (LSECs), Kupffer cells (KCs), and hepatic stellate cells (HSCs) into two adjacent fluid channels separated by a porous permeable membrane, mimicking its key structures and configurations. Such a liver chip was then demonstrated to have enhanced secretion of albumin, production of HGF, and metabolic activity of CYP450 under the exposure of shear flow, replicating primary immune responses. In our previous study (16), we proposed a simple method involving a liver microsystem to produce a mimicked SBF in the absence of vascular networks and successfully cultured HCs on a large, high-density scale. Despite the fact that these *in vitro* microfluidic liver-on-a-chip can provide cells with a microenvironment similar to that *in vivo*, simulating the anatomy, physiology and function of the liver, the tissue-tissue interfaces recreated in the state-of-the-art liver-on-a-chip platforms are essentially the orderly co-culture of diverse cells based on scaffolds such as porous membranes, which is detrimental to later organ transplantation research. Besides, there is no liver-on-a-chip that can enable genuine vascular networks. As such, further studies on the liver-on-a-chip remain in dire need.

In this study, we designed a tri-vascular liver-on-a-chip (TVLOC) comprising a hepatic artery, a portal vein, and a central vein. HCs and HSCs are hierarchically wrapped in bilayer microspheres to form liver microstructure, and the microspheres were surrounded with endothelial cells to generate a vascular network. Such a design is aimed at reconstructing liver lobules *in vitro* and providing cells with a substance concentration gradient to imitate *in vivo* microenvironment. Firstly, the design of TVLOC was proposed and the distributions of culture medium flow field and nutrient concentrations (oxygen and glucose) thereof were analyzed theoretically. Secondly, a high-throughput bilayer microsphere generating microsystem based on the coaxial confocal principle was developed. Lastly, cell viability and vascular network growth were observed and analyzed by means of experiments. This work will not only help predict drug hepatotoxicity and drug metabolism, but also facilitate the study of tumor invasion mechanisms *in vitro*.

## Materials and methods

### Materials and Instruments

The animal study was reviewed and approved by the Hefei University Ethics Committee. Type I collagen was prepared from the tail tendons of rats. Sodium alginate, 75% alcohol, glacial acetic acid, sodium chloride, disodium hydrogen phosphate, sodium dihydrogen phosphate, potassium dihydrogen phosphate, anhydrous sodium carbonate, bovine serum albumin (BSA), 4% paraformaldehyde, Triton X-100, Tween 20 were all purchased from Sangon Biotech Co., Ltd. (Shanghai, China). Beakers, ophthalmic Scissors, and tweezers were all obtained from the school reagent library; primary antibodies and fluorescent-labeled secondary antibodies were purchased from Wuhan Sanying Biology Technology Co., Ltd. Inverted fluorescence microscopes (IX53; Olympus, Japan), CO2 incubators (Thermo 3131), and computer-controlled microfluidic pumps (Harvard Apparatus PUMP33; Harvard Instruments, USA) were employed in this study.

### Design and Fabrication of the TVLOC

**Figure 1:**
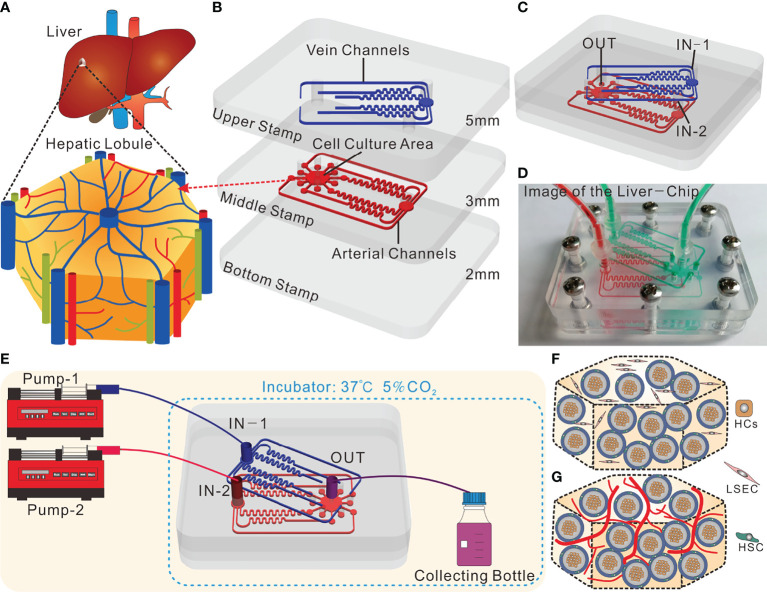
Design and operation of the tri-vascular liver-on-a-chip (TVLOC). **(A)** Schematic of the *in vivo* hepatic lobule; **(B)** Schematic components of the TVLOC; **(C)** Schematic of the assembled TVLOC; **(D)** The physical picture of the assembled TVLOC; **(E)** Schematic of the TVLOC operation system; **(F)** Schematic of the cultivation at early stage in the culture area; **(G)** Schematic of the formation of vascularized liver tissue in the culture area.

The core concept of the liver-on-a-chip designed in this study lies in the **introduction** of the tri-vascular system, which imitates the hepatic artery, hepatic portal vein and central vein, respectively (**Figure 1A**). This liver-on-a-chip microsystem was composed of three polymethyl methacrylate (PMMA) stamps engraved with microchannels. The dimensions of such PMMA stamps were as follows: both the length and width were 52 mm×45 mm; the thicknesses of the top stamp, the middle stamp and the bottom stamp were 4 mm, 3 mm and 2 mm, respectively (**Figure 1B**). This system was fabricated by Qingsheng Information Technology Co., Ltd., Hefei, China. The top stamp was engraved with six fluid microchannels, wherein in an attempt to ensure that the flow resistance of each microchannel was uniform, and as per Kirchhoff’s Laws of current and voltage, these microchannels were designed to possess identical lengths from the inlet to the outlet. In contrast, apart from six fluid microchannels with the same flow resistance, the middle stamp harbored a hexagonal cell culture area. Plus, each fluid microchannel on the upper stamp had a corresponding through hole on the middle stamp, allowing the fluid to enter the cell culture area (**Figure 1B**). In a bid to arrive at the real-time observation, the bottom layer should be made from a transparent and thin PMMA stamp. However, the PMMA stamp, if too thin (1mm), was readily subjected to deformation, and thus did harm to the liquid sealing; and if too thick, could result in the failure of real-time observations owing to the limited height of the microscope. As such, the thickness of the bottom stamp was selected to be 2 mm.

The assembled TVLOC was schematically displayed in **Figure 1C**. During the operation, the venous medium from the inlet (IN-1) traveled through the six microchannels on the top stamp, and subsequently entered the hexagonal cell culture zone *via* the six through holes carved on the middle stamp, wherein the upper ends of such through holes were connected with the six output ends of the microchannels, and the lower ends thereof were connected to the cell culture zone. By contrast, the arterial medium from the inlet (IN-2) moved through the six microchannels on the middle stamp before entering the cell culture zone. Upon converging in the cell culture area, the arterial medium and the venous medium formed substance concentration gradients and ultimately flowed out from the outlet (OUT). The physical picture shown in **Figure 1D** represented the assembled TVLOC perfused with red and green liquids, which represented the arterial medium and the venous medium, respectively. It can be seen that the liquids of distinct colors moved in their respective microchannels, converged in the cell culture area, and eventually were drained in the central vein. After being assembled, this system was fixed with stainless steel screws.

### Set-up of the TVLOC System

As depicted in **Figure 1E**, the experimental operation platform for the TVLOC microsystem primarily consisted of two microinjection pumps and the TVLOC. DMEM high-glucose complete medium (4.5 g/L glucose) contained in the syringe on pump 1 entered into the chip from IN-1 by virtue of the driving force of pump 1, moved through the six microchannels on the upper stamp followed by entering the hexagonal cell culture zone on the middle stamp *via* the corresponding through holes. In the meantime, DMEM low-glucose complete medium (1 g/L glucose) contained in the syringe on pump 2 flowed into the chip from IN-2 and then entered the hexagonal cell culture zone *via* the six microchannels on the middle stamp. After converging in the cell culture area, these two culture media flowed into the liquid collecting bottle through the outlet OUT, with both the pumps operated at a flow rate of 200 μL/h. During cell culture, 200 μL samples were taken from the liquid collecting bottle every 24 h to be used to analyze and detect the concentrations of metabolites therein. HCs, HSCs and LSECs (the three cell lines were obtained from ATCC) in the cell culture zone were orderly distributed in three dimensions (**Figure 1F**), with HCs embedded in the inner layer of the bilayer microspheres, HSCs located at the outer layer, and LSECs encapsulated in the collagen outside the microspheres. After being cultured for a period of time, LSECs would form a vascular network-like structure, thereby resulting in the formation of vascularized liver tissue (**Figure 1G**).

### Set-up of Experimental Platform for the Microsphere-generating System

**Figure 2:**
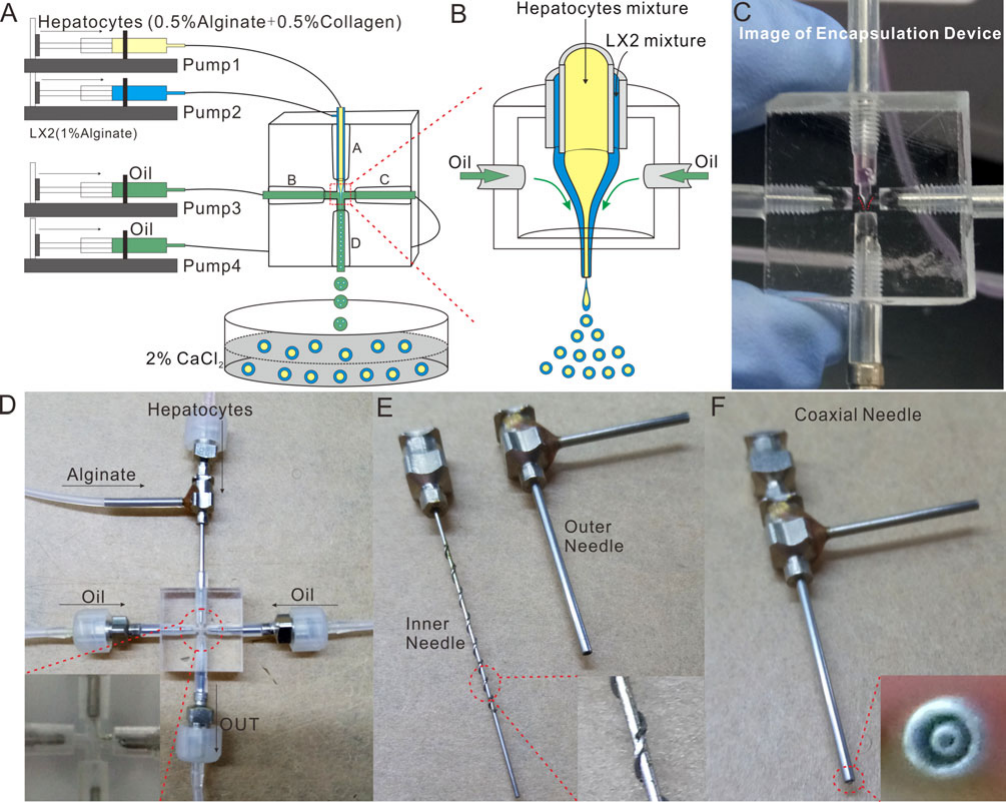
Design and Fabrication of the Microspheres-generating Chip. **(A)** Schematic of the bilayer microspheres-generating system; **(B)** Schematic of focusing the inner and outer phases to be cone-shaped; **(C)** The physical picture of focusing the inner and outer phases to be cone-shaped; **(D)** The physical picture of the microspheres-generating chip; **(E)** The exploded view of the coaxial needle; **(F)** Image of the assembled coaxial needle.

As illustrated in **Figure 2A**, the experimental operation platform for the microsphere-generating system was mainly composed of the core device for microsphere formation, silicone tubes and four microinjection pumps. Specifically, the syringe on pump 1 was loaded with the mixture of 0.5% collagen and HCs-laden 0.5% sodium alginate, the syringe on pump 2 contained HSCs (LX2 cells)-embedded 1% sodium alginate, and the syringes on pumps 3 and 4 were filled with oil for shearing liquid. During the operation, the flow velocities used by pumps 1 and 2 were both 2 mL/h, and those adopted by pumps 3 and 4 were identical and set to be in the range of 20-60 mL/h. Herein, the oil was referred to as the focusing phase, while the liquids flowing in the inner needle and the outer needle were named the inner phase and the outer phase, respectively. The focusing phase focused the inner and outer phases to be in a cone shape (**Figures 2B, C**), which can be controlled by the flow velocity of the focusing phase.

The core device for microsphere formation (i.e. the microsphere-generating chip) was made in accordance with the principle of compound flow focusing, and primarily composed of a coaxial needle, three ordinary needles and a cross-shaped chip (**Figure 2D**). The coaxial needle consisted of an inner needle (inner diameter: 0.41 mm, outer diameter: 0.71 mm) and an outer needle (inner diameter: 1 mm, outer diameter: 1.48 mm) (**Figure 2E**). The inner needle was 0.2 mm longer than the outer needle, and these two needles were integrated through a perforated silicone plug (**Figure 2F**). To guarantee the coaxiality of the needles, a plurality of metal wires were welded on the outer wall of the inner needle. The cross-shaped chip, made from PMMA, was transparent and thus enabled the real-time observation of fluid flow under the microscope. With a dimension of 20 mm × 20 mm × 10 mm, the cross-shaped chip was provided with a round hole on each of its four sides. Such four holes possessed an initial diameter of 3 mm, became narrower (0.3 mm) as they extended towards the center of the chip, and then formed a criss-cross connected with each other there, wherein the peripheral round hole was used to fix the needle, and the central criss-cross part was where the microspheres were produced by shearing (Qingsheng Information Technology Co., Ltd., Hefei, China).

### Preparation of Rat Tail Collagen

Collagen used herein was derived from rat tails. The detailed preparation procedures were as follows (17): rat tails were soaked in 75% alcohol for disinfection before their skin was ripped off. Tail tendons were then pulled out after the tailbones were broken in sections. Subsequently, the thus-obtained tail tendons were sterilized and cut into pieces, followed by being soaked in the sterile 0.1% acetic acid and placed at 4 °C for about 7 days until fully dissolved. After being subject to lyophilization, 10 mg/mL collagen was formulated with 0.1% glacial acetic acid and stored at 4 °C for later use.

### Loading of Cells into the TVLOC Microsystem

Liver sinusoidal endothelial cells (LSECs) at a final concentration 2×10^4^ cells/mL were firstly mixed with the prepared extracellular matrix (ECM) consisting of the neutralized collagen (final concentration: ~3 mg/mL), bovine fibrinogen (final concentration: ~2.5 mg/mL) and thrombin (final concentration: ~1.5 U/mL) (Of note, this step should be conducted on ice). Following that, the LSECs-laden ECM solution was mixed with the bilayer microspheres (total number: roughly 1×10^3^) at a volume ratio of 1:1, and a 50 μL mixture was then pipetted to the cell culture area on the TVLOC. Finally, the TVLOC was transferred to an incubator for 30 min. After the collagen was completely solidified, the TVLOC was connected with the micropump and placed in the incubator for culture (see **Figures 1F, G**). It is worth noting that HCs, HSCs and LSECs were all cultured in DMEM medium (Gibco) supplemented with 10% fetal bovine serum (Lonsera) and 1% penicillin-streptomycin (Gibco) at 37°C and 5% CO_2_ before being used within passage 4-12.

### Immunofluorescence Staining

For immunofluorescence assay, cells were fixed with 4% paraformaldehyde for 20min and washed with PBST three times. After being permeabilized in 0.1% Triton X-100 at room temperature (RT) for 7 min and washed with PBST three times, cells were blocked with 2% BSA in PBS at RT for 2h, followed by incubation with the primary antibody at RT for 2 h or at 4 °C overnight and then with the secondary antibody in the dark at RT for 40 min. Ultimately, cells were washed with PBST three times once again and then observed under an inverted fluorescence microscope (Olympus IX73).

### Cell Viability Assay

To assess the viability of cells during the culture period, a standard Live/Dead assay kit (Invitrogen, USA) containing Hoechst 33342 and propidium iodide (PI) (18, 19) was employed pursuant to the product instruction. Briefly, cells were stained with 10 μg/mL Hoechst 33342 and 10 μg/mL PI for ~30 min at 37°C. The individual blue cells (viable) and red cells (dead) were counted by Image J.

### Assessment of Albumin and Urea Production

The concentrations of urea and albumin in the samples collected from the liquid collecting bottle were analyzed using clinical biochemistry methods. To be specific, albumin secretion and urea production were measured by the Beckman Coulter AU480 biochemistry analyzer. The rates of change in the absorbance at 600 nm and 340 nm, which are proportional to the albumin and urea concentration, respectively, were recorded. In addition, to recheck the accuracy of the measured data, urea and albumin were further detected for their concentrations at 450 nm by ELISA.

### Statistical Analysis

Data were expressed as the means ± standard deviation. P-values were analyzed using the two-tailed unpaired Student’s t test. The significance level was indicated as * for P<0.05, ** for P<0.01, and N.S. for no significance.

## Modeling

In our TVLOC, the two inlets simulated the hepatic artery and portal vein respectively, and the outlet mimicked the central vein. Considering that the convergence of the hepatic artery and portal vein would result in concentration gradients of nutrients, to further figure out such gradients, a model was established using COMSOL Multiphysics software (Burlington, MA, USA) based on the following hypothesises: (1) the effect of cell metabolites on the flow velocity is negligible; (2) The flow in all areas conforms to Newton’s theory; (3) the properties of the medium (such as density and viscosity) are the same as those of water at 37°C; and (4) the effect of cells on the porosity of ECM is negligible.

### Geometric Model

**Figure 3:**
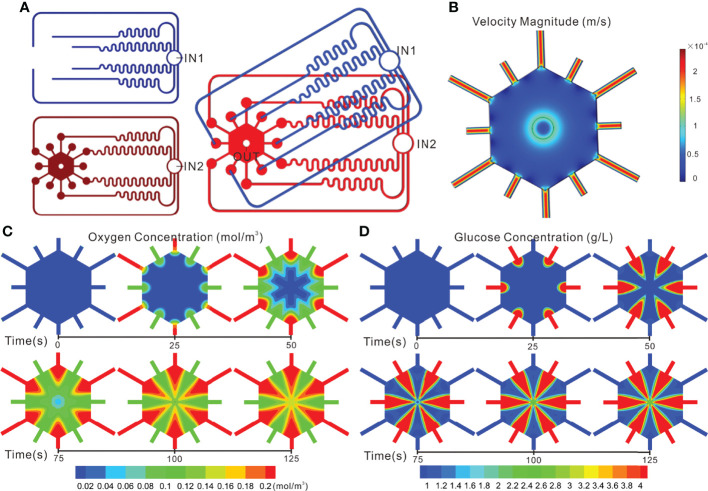
Simulation of flow and concentration distribution profiles in the TVLOC. **(A)** The top view of the geometric model; **(B)** Flow distribution in the TVLOC; **(C)** The distribution of oxygen concentration in the TVLOC; **(D)** The distribution of glucose concentration in the TVLOC.

The geometric model established herein is a three-dimensional one, the top view of which was exhibited in **Figure 3A**. This model was divided into three parts: the fluid area on the top stamp, the fluid area and the cell culture area on the middle stamp, wherein both of the fluid areas were utilized to observe whether the fluids from the inlets could be evenly distributed to the corresponding output ends, and then to the cell culture area. Additionally, the fluid combination is partly aimed at observing the concentration diffusion of oxygen and glucose in the cell culture area.

### Fluid Equation

The fluids in the TVLOC adopted the mode of laminar flow, and thus were described by the Naiver-Stokes equation (14, 20):


(1) $$\rho \left[\left(u\cdot \nabla \right)u\right]=\nabla \cdot \left[-pI+\mu \left(\nabla u+{\left(\nabla u\right)}^{T}\right)\right]$$

Here, *ρ*is the fluid density (kg/m3), *μ*is the fluid viscosity (N s/m2), *u*is the fluid velocity vector (m/s), *p* is the pressure, ∇ is the del-operator, and superscript *T* denotes the matrix transpose.

### Concentration Equations

In terms of substance concentrations, herein, the oxygen and glucose concentrations were simulated respectively. Specifically, at the inlet of the hepatic artery, the oxygen partial pressure was set to 90 mmHg, and the concentration of glucose was set to 1.0 g/L, while at the inlet of the portal vein, the oxygen partial pressure was set to 30 mmHg, and the concentration of glucose was set to 4.5 g/L. Additionally, the diffusion coefficient of oxygen in water at 37 °C was 2.92×10^-9^ m2s^-1^ (21), and the diffusion coefficient of glucose was 9×10^-10^ m2s^-1^ (14). Diffusion was described by the following standard stationary convection-diffusion equations:

for oxygen,


(2) $$u_{o}\cdot \nabla c_{o}+\nabla \cdot \left(-D_{o}\nabla c_{o}\right)=-\nabla_{o}$$

for glucose,


(3) $$u_{g}\cdot \nabla c_{g}+\nabla \cdot \left(-\nabla_{g}\nabla c_{g}\right)=-\nabla_{g}$$

Here, the subscripts o and g represent oxygen and glucose, respectively, *c*represents the oxygen concentration (mol/m^3^), *D*is the diffusion coefficient (m^2^/s), *V*represents the oxygen consumption rate (mol/1/s), and *u* is the flow velocity (m/s). In the above two equations, the first and second items on the left represent the oxygen transport caused by convection and diffusion respectively. The oxygen partial pressure *P*is routinely indirectly replaced by oxygen concentration (22, 23). Besides, the relationship between oxygen concentration and oxygen partial pressure can be depicted by Henry’s law (24): c = α*P* (here *α*is the oxygen dissolution coefficient).

### Boundary Conditions

The velocity and pressure boundary conditions adopted in the simulation were as follows: at the inlets of the TVLOC, a uniform velocity was utilized, and the perfusion flow rate of the culture medium was given; at the outlet, the pressure was set to 0 Pa, and the concentration condition, ∂*c*/∂*n* = 0 was adopted.

### Numerical Simulation

In this study, as mentioned above, COMSOL Multiphysics software was employed to conduct simulation. Firstly, to render the calculation more realistic, a three-dimensional geometric model, just as shown in **Figure 3A**, was established in the software. Secondly, relevant parameters and boundary conditions were set. Thirdly, the meshes were divided, and finer meshes were used herein. Fourthly, numerical simulations were performed to obtain the fluid distribution in the TVLOC and the distribution of oxygen and glucose concentrations in the mixed region. Of note, in the calculation process, to quickly converge, the velocity field was calculated before the concentration field was assessed.

## Results and discussion

### On-chip Flow Distribution

The design of the fluid channels was in accordance with Kirchhoff’s Laws of current and voltage. To ensure identical fluid resistance, the six fluid microchannels on the upper stamp and those on the middle stamp must be kept consistent in length as far as possible. With the fluidic behavior of laminar flow adopted in the numerical simulation, the fluid distribution on the TVLOC was demonstrated in **Figure 3B**. When the arterial and venous inlet velocities were both 200 μL/h, the flow velocities at the six microchannel output ends on the upper stamp are 2.1705×10^-4^, 2.1716×10^-4^, 2.1780×10^-4^, 2.1751×10^-4^, 2.1734×10^-4^, and 2.1764×10^-4^ m/s, respectively, and the flow velocities at the six microchannel output ends on the middle stamp are 2.1727×10^-4^, 2.1796×10^-4^, 2.1743×10^-4^, 2.1764×10^-4^, 2.1749×10^-4^, and 2.1799×10^-4^ m/s, respectively. Consequently, on account of the relatively uniform flow velocity and close flow resistance in the twelve microchannels, the culture medium therein would be less inclined to flow out from just one or several channels, thereby ensuring the flow of the medium into the hexagonal cell culture area through each of these microchannel output ends around it. It can be explicitly seen that the cell culture area got a flow velocity of 0.5-5×10^-5^ m/s and thus produced a relatively low flow shear force. As such, cells cultured in this area would not suffer from the damage of shear force and could be supplied with adequate nutrients.

Noticeably, the above simulation was based on the initial cell density. When just loaded into the culture area, the cells were present at a low level, relatively dispersed, and failed to form cell clusters or vascular networks; therefore, the flow distribution in the culture area was relatively uniform. As the culture time went by, HCs would gradually form cell clusters, LSECs would link with each other to form a vascular network, and changes would necessarily occur to the flow distribution. Given that the in-depth analysis of the changes of fluids with culture time needs the establishment of extremely complicated models, in this work, merely a simple model was created to observe the initial flow changes in the culture area.

### On-chip Oxygen/glucose Concentration Profile

To better understand the distribution of substance concentration gradients in the cell culture area, COMSOL simulation software was used herein to mimic the distribution of oxygen and glucose concentration gradients. Now that the hepatic artery is rich in blood oxygen, the arterial medium was set to be a high-oxygen low-glucose one (oxygen level: 90 mmHg, glucose concentration: 1 g/L). By contrast, the hepatic vein is full of blood rich in nutrients from the digestive tract, spleen, among others; therefore, the venous medium was set to be a low-oxygen high-glucose one (oxygen level: 30 mmHg, glucose concentration: 4.5 g/L). Here, with the flow velocities at the inlets of the hepatic artery and portal vein set to be 200 μL/h, the changes of substance concentrations in the cell culture area over time were observed (**Figures 3C, D**). From the results, it can be clearly seen that remarkable concentration gradient distribution in the cell culture area occurred to both the oxygen and glucose. Specifically, the initial concentrations of oxygen and glucose were at low levels without the formation of concentration gradients. After 100 s, both of them developed a stable concentration gradient.

Accordingly, this TVLOC was capable of providing a 3D cell growth condition imitating the liver microenvironment, and thus could be used for the subsequent study on the functional mechanism of liver zonation. It is worth mentioning that, in the practical experiments below, the effect of oxygen concentration gradients on hepatocyte function was not investigated due to no dissolved oxygen controlling system available. In our future work, an oxygen concentration regulating chip will be designed to precisely control the dissolved oxygen concentration of the arteriovenous culture medium so as to further mimic the microenvironment *in vivo*.

### Effect of Preparation Speed on the Size of Bilayer Microspheres

**Figure 4:**
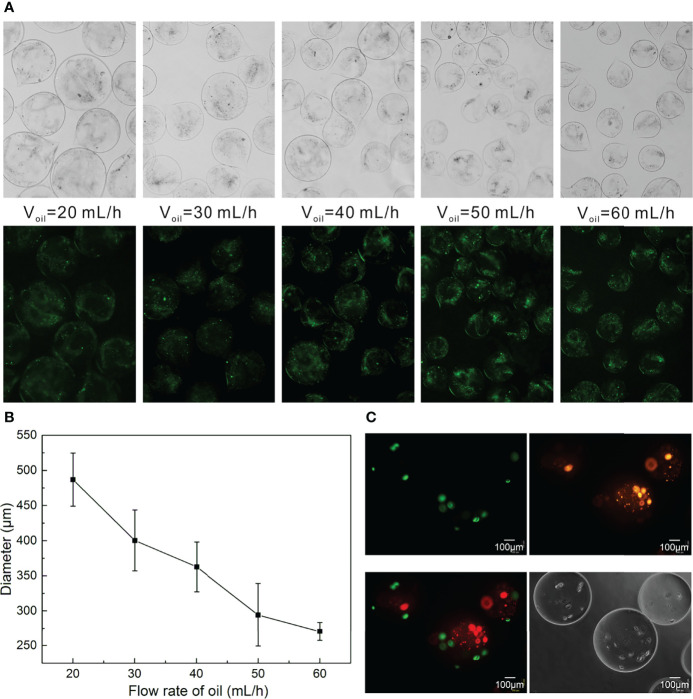
Preparation of Bilayer Microspheres. **(A)** Morphology diagram of the size of the microspheres as a function of the oil flow rate; **(B)** Statistical diagram of the size of the microspheres as a function of the oil flow rate; **(C)** Morphology images of the bilayer microspheres.

The morphology and size distribution of bilayer microspheres were closely associated with the fluid velocity adopted during the preparation. In this respect, **Figures 4A, B** indicated the relationship between the size of the bilayer microspheres and the flow rate of the focusing oil phase. In the case that the inner and outer flow velocities in the coaxial needle were set to be 2 mL/h, the size of microspheres gradually decreased with the increase of the flow rate of the oil phase. Here, for the convenience of observation, the inner and outer sheath fluids in the coaxial needle were added with green fluorescent particles and sodium alginate, respectively. As illustrated in **Figure 4A**, the green fluorescent particles were well wrapped in the microspheres without leakage, and as the flow rate of the oil phase increased, these microspheres tended to get smaller and possess better uniformity. The specific changes obtained through measurement and statistical analysis were as follows (**Figure 4B**): when the inner and outer flow velocities in the coaxial needle were both 5 mL/h, and the flow rate of the oil phase was 20 mL/h, the diameter of these microspheres was approximately 487 μm. When the flow rate of the oil phase rose to 60 mL/h, the microspheres were reduced roughly by half in size, with the diameter being only 270 μm.

It should be readily understood that the size of the microspheres could also be adjusted by regulating the inner and outer flow velocities in the coaxial needle. Likewise, the liquid viscosity also posed impacts on the size of the microspheres, which can be modulated as required. In addition to the aforementioned control on the size of such microspheres, the shell thickness of microspheres could be regulated by adjusting the velocity ratio of the inner and outer sheath fluids. Since the size of the microspheres affects the transport of oxygen and nutrients, this work merely took into account the relationship between the size of microspheres and the flow velocity of the oil phase. In an effort to ensure the survival and proliferation of cells in microspheres, the bilayer microspheres prepared herein harbored a diameter of about 270 μm. In the follow-up work, the outer shell thickness of the microspheres will be further optimized to make the shell as thin as possible, which is more conducive to the simulation of space of Disse. Plus, the bilayer microspheres prepared in this paper are not round enough, which is likely owing to the different viscosities of the inner and outer sheath fluids. In future work, to further beef up the quality and yield of such microspheres, adjusting the viscosities of the inner and outer sheath fluids, and submerging the outlet into the CaCl_2_ solution, among others, should be given priority.

### Cell Distribution in the Bilayer Microspheres

To observe the distribution of HCs and HSCs in the bilayer microspheres, HCs and HSCs were pre-stained with DiI (Orange) and DIO (green) prior to the formation of the microspheres. As demonstrated in **Figure 4C**, HCs (HepG2 cells) were colored green and located at the inner layer of the microspheres, while HSCs (LX2 cells) were colored and embedded in the outer layer of the microspheres. Of note, in the inner sheath fluid, HepG2 cells were suspended in a mixture of collagen and sodium alginate (0.5 mg/mL for collagen and 0.5 mg/mL for sodium alginate) at a final density of 6 × 10^6^ cells/mL. In the outer sheath fluid, LX2 cells were suspended in 1% sodium alginate solution at a final density of 5 × 10^5^ cells/mL. Such a cell density ratio of roughly 10:1 was set based on the actual ratio of HCs to HSCs *in vivo*.

### Off-chip Culture of the Bilayer Microspheres

**Figure 5:**
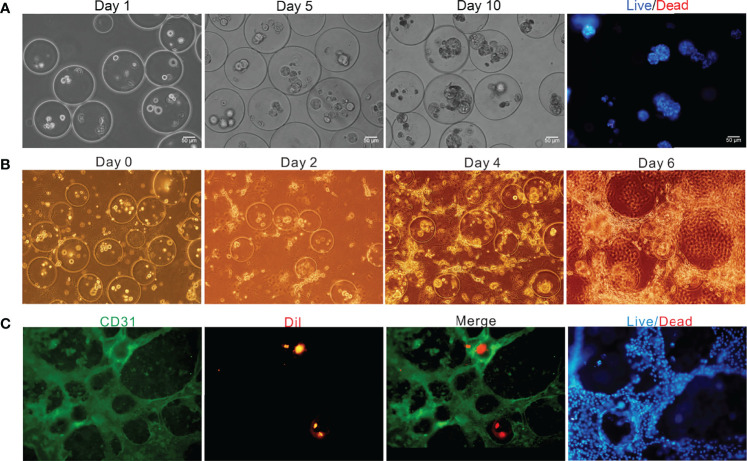
Off-chip and on-chip culture of the bilayer microspheres. **(A)** Off-chip cell growth in the microspheres as a function of time; **(B)** Brightfield image of the vascularized live microtissue cultured on chip; **(C)** Fluorescence image of the vascularized live microtissue cultured on chip.

To better observe the changes of cell growth in the bilayer microspheres, herein, the bilayer microspheres were firstly cultured in a Petri dish, and the morphological changes of the cells were observed every day. Results from **Figure 5A** showcased that initially in the bilayer microspheres, most of the cells were singly distributed. With the extension of culture time, the volume of cell clusters gradually increased, and the cells had formed a compact tissue structure by Day 10, with the size of microstructure reaching 120 μm. Using Hochest/PI staining, almost all the cells in the microspheres were shown to be alive. Notably, considering that the diffusion distance of oxygen and nutrients is less than 200 μm, the bilayer microspheres possessing an average diameter of roughly 270 μm were employed in this experiment. These small microspheres could allow for sufficient material transport, thereby enabling the survival and proliferation of cells encapsulated in the microspheres.

### On-chip Co-culture of Cells

After being generated and verified to be feasible as mentioned above, the bilayer microspheres were mixed in equal volume with the prepared ECM containing LSECs (the final concentrations of type I rat tail collagen and bovine fibrinogen were roughly 1.5 mg/mL and 1.25 mg/mL, respectively, and the cell density of LSECs was about 2×10^4^ cells/mL) and then loaded into the cell culture area of the TVLOC for solidification, followed by long-term culture. The formation of vascularized liver microstructure was depicted in **Figure 5B**. It can be observed that in the first four days of the co-culture, LSECs gradually elongated and linked with each other to form the preliminary structure of vascular network. On Day 6, a distinct vascular network was ultimately formed. The bilayer microspheres were wrapped with the vascular network, thereby resembling the liver microtissues *in vivo* to some extent.

To further demonstrate the vascularization of the liver microtissue, LSECs were labeled with CD31, and HCs in the microspheres were pre-stained with DiI in advance. As validated by the immunofluorescence staining shown in **Figure 5C**, a vascular network was resulted from the connection of LSECs with each other, and the microspheres were then surrounded by the vascular network, forming a vascularized liver tissue. Additionally, the analysis of cell survival through staining with Hochest/PI revealed that nearly all the cells in the cell culture area were alive, indicating the superb growth and proliferation ability of the vascularized liver microtissue in the TVLOC. Here, it’s worth noting that, DiI instead of specific antibodies was adopted to pre-stain HCs due to the poor specificity of the antibodies available; however, over a long period of cultivation, the fluorescence of DiI was gradually quenched, resulting in less distribution of liver microtissue observed under the microscope field than in the actual situation. To address such an issue, in the follow-up work, preparing stably transfected cell lines with fluorescence should be taken into consideration.

### On-chip Analysis of Synthetic and Metabolic Functions of HCs

**Figure 6:**
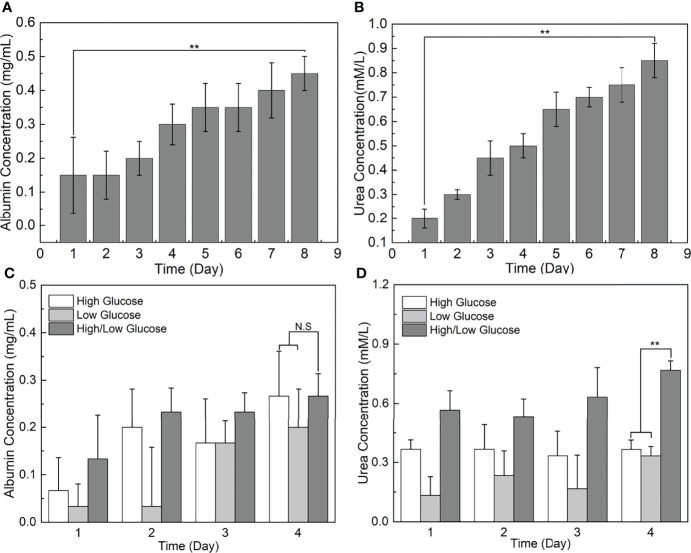
Liver-specific metabolite concentrations as a function of time. **(A)** The change of albumin concentration in the medium over time; **(B)** The change of urea concentration in the medium over time; **(C)** The effect of glucose concentration gradients on the albumin synthesis of HCs; **(D)** The effect of glucose concentration gradients on the urea metabolism of HCs. **p < 0.01.

To further assess the performance of our TVLOC, the changes of liver-specific metabolite concentrations over time were studied herein. As is well-known, albumin is one of the liver-specific biomarkers, and urea is a primary measure of hepatocyte functionality. Therefore, in the present study, albumin synthesis and urea production were detected to analyze the viability of HCs in the TVLOC. During the long-term (8-day) co-culture of HCs, HSCs and LSECs, as depicted in **Figures 6A, B**, both the concentrations of the albumin and urea increased with time, suggesting that in this TVLOC, these cells could grow and proliferate stably, and the specific functions of HCs could be strongly maintained for a long time.

It is remarkable, however, that all the cells used in this work were cell lines, primary cells should be considered to be used for verifying the effectiveness of this system in future research. In this connection, how to efficiently separate all the four types of cells from the primary cells remains elusive, and hence the experimental conditions such as the antibody concentration in flow sorting need to be explored. Despite this, the excellent performance of the TVLOC could be highly expected. Furthermore, by way of example, the performance of this TVLOC could be further improved by adjusting the concentrations of collagen and sodium alginate to control the stiffness of the ECM and providing the culture medium with hormone gradients.

### Effect of Glucose Concentration Gradients on Hepatocyte Function

In light of the fact that glucose metabolism is one of the vital functions of the liver, and the external glucose concentration inevitably poses a certain impact on the function of HCs, this study adopted the TVLOC to assess the effect of different glucose concentrations on HCs. To be specific, the changes of liver function over time under three different sugar concentrations, namely high sugar, low sugar and high-low sugar, were investigated. From **Figures 6C, D**, it can be seen that, after 4 days of culture, no significant difference occurred to albumin concentration in the cases of the three sugar concentrations, which was most likely owing to the non-interference between glucose metabolism and protein synthesis. That is, the external glucose concentration failed to affect the albumin synthesis. By contrast, the urea nitrogen synthesis capacity of HCs under the condition of glucose concentration gradients (i.e. the high-low sugar concentration) was enormously higher than that of HCs under the high or low glucose condition. We speculate that since HCs in different regions exert different liver functions due to the existence of liver zonation along the hepatic sinusoid, the presence of glucose concentration gradients here might stimulate the generation of corresponding hepatocyte zonation and result in a microenvironment closer to *in vivo* one, thereby improving the corresponding urea synthesis function of HCs.

## Conclusions

Currently, the rapid development of organ-on-chips has shed light on human organ reproduction (25, 26). Such *in vitro* models pose a subversive impact on conventional 2D cell culture, and play central roles as supplements or alternatives to animal models in drug screening and toxicity testing.

In the current study, we proposed a tri-vascular liver-on-a-chip (TVLOC) reconstructing the tissue-tissue interfaces based on bilayer microspheres. This TVLOC primarily contains a hepatic artery fluid supply channel, a portal vein fluid supply channel and a hepatic lobular hexagonal cell culture area. With HCs located at the inner layer and HSCs cells embedded in the outer layer, HCs and HSCs were wrapped in the bilayer microspheres at a cell density ratio of 10:1 (based on the actual ratio *in vivo*). In this way, the tissue-tissue interfaces were reconstructed by virtue of the bilayer microspheres, and the layer of hydrogel where HSCs are encapsulated simulated the space of Disse. In addition, LSECs formed a vascular network surrounding the bilayer microspheres, thereby creating a vascularized liver microtissue in the cell culture area. To analyze the performance of this TVLOC, firstly, a theoretical model was established to observe the fluid distribution and the oxygen/glucose concentration distribution in the TVLOC respectively. Secondly, the relationship between the size of microspheres and the flow rate of the oil phase was analyzed to select microspheres of appropriate size. As the oxygen diffusion distance does not exceed 200 μm, the size of the microspheres to be used shall not exceed 400 μm. Lastly, to verify the performance of the TVLOC, the concentrations of metabolites (albumin and urea) in the culture medium were detected. It was found that the concentrations of these two substances increased with time, indicating that this chip can well maintain the specific function of hepatocytes. Collectively, the TVLOC designed in this work provides insights into further development of the liver-on-a-chip and holds great potential in drug screening and evaluation. Besides, the TVLOC, as a vascularized liver chip, can be used to research the progress of tumor metastasis because the tumor metastasis typically occurs through blood vessels.

## Data Availability Statement

The original contributions presented in the study are included in the article/supplementary material. Further inquiries can be directed to the corresponding authors.

## Ethics Statement

The animal study was reviewed and approved by Hefei University Ethics Committee.

## Author Contributions

Conceptualization, JL and HL; methodology, JL and MZ; software, CF; formal analysis, HL; investigation, HL; resources, FS; data curation, JL and CF; writing—original draft preparation, JL; writing—review and editing, HL; supervision, FS; project administration, CF. All authors have read and agreed to the published version of the manuscript.

## Funding

This research was funded by the Natural Science Foundation of Anhui Province (grant number 2008085QC116), the University Natural Sciences Research Project of Anhui Province (grant number KJ2019A0824), the Talent Research Foundation of Hefei University 20RC41 and Taishan Young Scholars Program of Shandong Province (grant number tsqn20161049).

## Conflict of Interest

The authors declare that the research was conducted in the absence of any commercial or financial relationships that could be construed as a potential conflict of interest.

## Publisher’s Note

All claims expressed in this article are solely those of the authors and do not necessarily represent those of their affiliated organizations, or those of the publisher, the editors and the reviewers. Any product that may be evaluated in this article, or claim that may be made by its manufacturer, is not guaranteed or endorsed by the publisher.
